# Analysing the reported incidence of COVID-19 and factors associated in the World Health Organization African region as of 31 December 2020

**DOI:** 10.1017/S095026882100193X

**Published:** 2021-08-16

**Authors:** Franck Mboussou, Benido Impouma, Bridget Farham, Caitlin M. Wolfe, George Sie Williams, Roland Ngom, Milse Nzingou, Aziza Merzouki, Erol Orel, Yahaya Ali Ahmed, Olivia Keiser, Matshidiso R Moeti

**Affiliations:** 1 World Health Organization, Regional Office for Africa, Brazzaville, Congo; 2 Institute of Global Health, University of Geneva, Geneva, Switzerland; 3 College of Public Health, University of South Florida, Tampa, Florida, USA

**Keywords:** African region, COVID-19, incidence

## Abstract

This study analysed the reported incidence of COVID-19 and associated epidemiological and socio-economic factors in the WHO African region. Data from COVID-19 confirmed cases and SARS-CoV-2 tests reported to the WHO by Member States between 25 February and 31 December 2020 and publicly available health and socio-economic data were analysed using univariate and multivariate binomial regression models. The overall cumulative incidence was 1846 cases per million population. Cape Verde (21 350 per million), South Africa (18 060 per million), Namibia (9840 per million), Eswatini (8151 per million) and Botswana (6044 per million) recorded the highest cumulative incidence, while Benin (260 per million), Democratic Republic of Congo (203 per million), Niger (141 cases per million), Chad (133 per million) and Burundi (62 per million) recorded the lowest. Increasing percentage of urban population (*β* = −0.011, *P* = 0.04) was associated with low cumulative incidence, while increasing number of cumulative SARS-CoV-2 tests performed per 10 000 population (*β* = 0.0006, *P* = 0.006) and the proportion of population aged 15–64 years (adjusted *β* = 0.174, *P* < 0.0001) were associated with high COVID-19 cumulative incidence. With limited testing capacities and overwhelmed health systems, these findings highlight the need for countries to increase and decentralise testing capacities and adjust testing strategies to target most at-risk populations.

## Introduction

Since early January 2020, the coronavirus disease 2019 (COVID-19) has spread globally and was declared a pandemic by the World Health Organization (WHO) on 11 March 2020 [1]. The COVID-19 is an acute respiratory illness caused by the severe acute respiratory syndrome coronavirus 2 (SARS-CoV-2), first reported in December 2019 in Wuhan, China [2]. The virus spreads by close person-to-person contact, mainly via respiratory droplets or aerosols produced when an infected person coughs, sneezes, sings, exercises or talks [2].

Twelve months into the pandemic, the African region, one of the six WHO regions [3], remains the second least affected, accounting for 2.4% of confirmed cases and deaths globally [4]. As of 3 January 2021, over 83.3 million COVID-19 cases and 1.8 million deaths were reported globally, of which 1.9 million cases and over 43 000 deaths were recorded in the WHO African region [4] which is comprised of 47 of the 54 countries on the African continent, constituting its Member States [5]. The relatively low number of COVID-19 cases and deaths could partly be explained by insufficient testing, a comparatively young population, favourable climate and possible cross-immunity [6–8].

Despite the low caseload, the COVID-19 pandemic remains a major threat to public health security in the WHO African region. Countries in this region are characterised by heterogeneous income levels; 45% are classified as low-income, 54% middle-income and 4% high-income countries, as well as underfunded and fragile health systems [9, 10]. At the same time, most countries experience recurrent infectious diseases outbreaks. In 2018, 96 new disease outbreaks were reported across 36 of the 47 Member States with cholera, measles and yellow fever being the most commonly reported [11].

The response to these recurrent diseases has informed the overall coordination of the response to the COVID-19 pandemic in the WHO African region. Using its internal grading system, the WHO graded the pandemic at the highest level, requiring regional offices, with assistance from the WHO headquarters, to coordinate support to Members States in responding to the event, as per the WHO emergency response framework [12]. This is also the first time that the WHO Regional Office for Africa (WHO AFRO) had to provide technical assistance and coordinate international support to all Member States simultaneously.

In order to prioritise countries requiring support, WHO AFRO monitors a set of key epidemiological parameters, including new cases and deaths, case fatality ratio, healthcare worker infections and cumulative incidence. Monitoring the disease incidence at national and regional levels helps WHO AFRO track the evolution of COVID-19, assess the effect of different public health and social measures on the trajectory of the pandemic, identify hotspots, compare current trends in different countries and areas within countries, and allocate the resources required to support the ongoing response. The COVID-19 incidence is also one of the key indicators used to assign a transmission pattern and alert level to each Member State, as per the WHO guidance for implementing and adjusting public health and social measures in the context of the COVID-19 pandemic [13]. The difference in cumulative incidence between countries can be explained by a wide range of factors including but not limited to testing strategies, health system capacity to respond to COVID-19 and population density.

The objectives of this study were to compute the reported incidence of COVID-19 in each country of the WHO African region over time and identify the epidemiological and socioeconomic factors associated with COVID-19 incidence.

## Methods

We conducted a retrospective study using the most recent data on confirmed cases of COVID-19 reported by countries in the WHO African region between 25 February and 31 December 2020.

Key epidemiological and selected socio-economic factors from the literature were used to analyse factors that may be associated with the incidence of COVID-19 in countries from the WHO African region.

### Inclusion and exclusion criteria

Only cases meeting the COVID-19 case definition as per the most updated WHO technical guidance [14] and laboratory-confirmed COVID-19 cases using a reverse transcriptase polymerase chain reaction test or an antigen rapid diagnostic test approved by the WHO, and officially reported by Member States were considered for this study.

We included countries of the WHO African region that reported confirmed COVID-19 cases to the WHO from 25 February to 31 December 2020. Countries that did not formally submit reports on COVID-19 cases including reports of zero cases during the last 3 months were excluded. Among the 47 countries of the WHO African region, only Tanzania met the exclusion criteria.

### Data sources and measurement

We built a dataset that included the following variables: country, population, cumulative number of cases, cumulative number of tests performed, percentage of the population aged 15–64 years, population density, percentage of the population living in urban areas, universal health service coverage, health expenditure (as a per cent of gross domestic product), prevalence of diabetes, prevalence of obesity, crude death rate, incidence of human immunodeficiency virus (HIV) infections, incidence of tuberculosis, mortality rate attributed to exposure to unsafe WASH (water, sanitation and hygiene) services, and density of nursing and midwifery personnel.

Data were extracted from:

- The line list of COVID-19 cases maintained by the WHO AFRO based on notification by its Member States between 25 February and 31 December 2020 as per their obligations under the International Health Regulations (2005) [15] and the Integrated Disease Surveillance and Response Strategy (IDSR) [16]. Only laboratory-confirmed COVID-19 cases officially reported by Member States were recorded in this dataset. Data were summarised as a cumulative number of COVID-19 confirmed cases reported as of 31 December 2020 by each country. The cumulative incidence expressed as cases per million population was computed by dividing the cumulative number of confirmed cases by the total population.
- The World Bank Open Data (2019 revision) for population density (people per square kilometre land area), percentage of population aged 15–64 years, percentage of urban population (percentage of population living in urban areas), universal health coverage index (average coverage of essential services based on tracer interventions that include reproductive, maternal, new-born and child health, infectious diseases, non-communicable diseases and service capacity and access, among the general and the most disadvantaged population) and health expenditure (per cent of gross domestic product) by country [17].
- The WHO diabetes country profiles 2016 for the prevalence of diabetes and prevalence of obesity [18].
- World Mortality 2019 data for the crude death rate [19].
- The database on tests performed as of 31 December 2020, maintained by WHO AFRO based on country reports. The daily number of PCR tests performed by each country was recorded in this dataset. We computed the cumulative number of tests performed per 10 000 population by dividing the cumulative number of tests performed by the total population of each country.
- The World Health Statistics 2020 [20] for the incidence of HIV infections (number of new HIV infections per 1000 population), the tuberculosis incidence (number of new tuberculosis cases per 100 000 population), the mortality rate attributed to exposure to unsafe WASH services (expressed per 100 000 population) and the density of nursing and midwifery personnel (number of nursing and midwifery personnel per 1000 population).

### Data analysis

Using the dataset derived from all collected data, we performed the following analyses:

- Evolution of the COVID-19 incidence over time

We computed the COVID-19 incidence in each country of the WHO African region during six periods corresponding to time for surpassing the following benchmark in terms of cumulative confirmed cases: 1000, 100 000, 500 000, 1 million and 1.5 million of cumulative COVID-19 cases. The following periods were considered accordingly: 25 February–13 April 2020 (period 1), 14 April–31 May 2020 (period 2), 1 June–14 July 2020 (period 3), 15 July–23 August 2020 (period 4), 24 August–1 December 2020 (period 5) and 2 December–31 December 2020 (period 6).

- Multivariable analysis of factors associated with COVID-19 incidence

Our outcome of interest was the cumulative COVID-19 incidence. All other variables included in the dataset were selected as covariates. Covariates included the cumulative number of tests performed per 10 000 population, percentage of population aged 15–64 years, population density, percentage of urban population, universal health service coverage index, health expenditure, prevalence of diabetes, prevalence of obesity, crude death rate, HIV incidence, tuberculosis incidence, mortality rate attributed to exposure to unsafe WASH services and density of nursing and midwifery personnel.

Due to its flexibility in allowing for overdispersion, risk factors for cumulative COVID-19 incidence were fitted using negative binomial regression. We assumed that the negative binomial model was more appropriate than the Poisson model. The likelihood ratio test was used to test this assumption.

Both univariable and multivariable regression models were fitted. As a result of the univariable analysis, all covariates with a *P*-value <0.25 [21] were included in the initial multivariable model. A backward stepwise method was used with gradual deletion of variables with *P* ≥ 0.25. The strength of the association between each covariate and the dependant variable was measured by the *β* coefficients with a 95% confidence interval. The likelihood ratio test was used to test the goodness of fit of the final model.

Data were analysed using R version 4.0.3 [22] for statistical analysis and ESRI 2017 ArcGIS Pro 2.1.0 [23] for mapping.

## Results

### Cumulative incidence by country

**Fig. 1.**
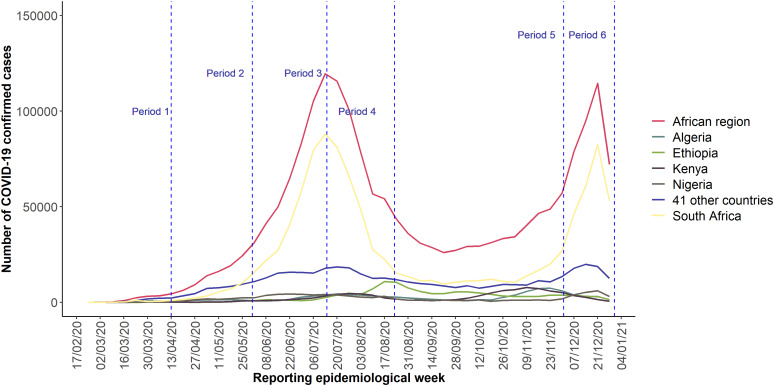
Distribution of COVID-19 confirmed cases by week of reporting in the WHO African region, top 5 countries and 41 other countries (data as of 31 December 2020). Period 1: 25 February–13 April 2020, Period 2: 14 April–31 May 2020, Period 3: 1 June–14 July 2020, Period 4: 15 July–23 August 2020, Period 5: 24 August–1 December 2020, Period 6: 2 December–31 December 2020.

Between 25 February 2020 and 31 December 2020, a total of 1 906 726 laboratory-confirmed cases of COVID-19 including 43 067 deaths were reported from the 46 countries of WHO African region included in this study, resulting in a case fatality ratio of 2.3%. The countries that reported the highest number of cases were South Africa (1 057 561), Ethiopia (124 264), Algeria (99 610), Kenya (96 458) and Nigeria (87 510). These countries accounted for 76.9% of reported cases during the study period. The distribution of confirmed cases reported weekly in the five most affected countries from the WHO African region is shown in Figure 1.

**Fig. 2.**
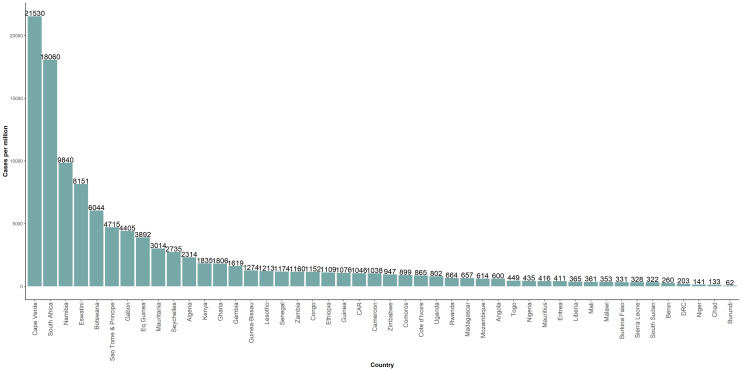
Cumulative number of COVID-19 cases per million population in 46 countries of the WHO African region, as of 31 December 2020.

The cumulative incidence as of 31 December 2020 in the WHO African region was 1846 cases per million population. The highest cumulative incidence was reported from Cape Verde (21 350 per million), South Africa (18 060 per million), Namibia (9840 per million), Eswatini (8151 per million) and Botswana (6044 per million). The lowest incidence was seen in Benin (260 cases per million), Democratic Republic of Congo (203 cases per million), Niger (141 cases per million), Chad (133 cases per million) and Burundi (62 cases per million). Figure 2 shows the distribution of countries by cumulative incidence as of 31 December 2020.

### Evolution of the cumulative incidence over time

During period 1 (25 February–13 April 2020), which lasted 46 days, the median incidence was 6 cases per million (range: 0–256). The median incidence then increased to 48 cases per million (range: 1–2227) during period 2 (14 April to 31 May 2020), 133 cases per million (range: 5–4507) during period 3 (1 June–14 July 2020), 156 cases per million (range: 2–5101) during period 4 (15 July–23 August 2020) and 224 cases per million (range: 18–13 245) during period 5 (24 August–1 December 2020), before decreasing to 89 cases per million (range 3–4459) during period 6 (2–31 December). The three countries with the highest incidence by period were (i) Mauritius (256 per million), Algeria (46 per million) and South Africa (39 per million) during period 1; (ii) Sao Tome and Principe (2227 per million), Equatorial Guinea (1278 per million) and Gabon (1253 per million) during period 2; (iii) South Africa (4507 per million), Cape Verde (2404 per million) and Equatorial Guinea (1944 per million) during period 3; (iv) South Africa (5101 per million), Cape Verde (3040 per million) and Eswatini (2383 per million) during period 4; (v) Cape Verde (13 245 per million), Botswana (3708 per million) and Namibia (3334 per million) during period 5; (vi) and South Africa (4459 per million), Namibia (3987 per million) and Eswatini (2528 per million) during period 6 (Table 1).

**Table 1.** COVID-19 incidence (cases per million population) in the African region by period from 25 February to 31 December 2020^a^

Country	25 February–13 April (Period 1)		14 April–31 May (Period 2)		1 June–14 July (Period 3)		15 July–23 August (Period 4)		24 August–01 December (Period 5)		02 December–31 December (Period 6)	
	Nb* cases	Incidence	Nb cases	Incidence	Nb cases	Incidence	Nb cases	Incidence	Nb cases	Incidence	Nb cases	Incidence
Algeria	1983	46	7411	172	10 822	251	21 244	493	42 692	992	15 458	359
Angola	19	1	67	2	455	16	1630	56	13 080	447	2302	79
Benin	35	3	208	17	1220	98	652	52	940	75	196	16
Botswana	13	5	25	10	361	147	909	371	9409	3841	4088	1669
Burkina Faso	515	25	368	18	155	8	300	15	1672	81	3818	185
Burundi	5	0	58	4	206	16	161	12	259	20	133	10
Cameroon	855	33	5049	195	10 153	392	2916	113	5647	218	2228	86
Cape Verde	10	18	425	773	1345	2446	1729	3144	7307	13 287	1024	1862
CAR	11	2	1058	223	3293	694	328	69	231	49	42	9
Chad	23	1	767	48	94	6	102	6	714	45	413	26
Comoros			106	125	215	253	96	113	196	230	152	179
Congo	74	14	537	100	1747	325	1492	277	1924	358	426	79
Cote d'Ivoire	654	25	2179	85	10 204	397	4550	177	3791	147	872	34
DRC	241	3	2954	34	4968	57	1679	19	3145	36	4671	54
Eq Guinea	41	30	1733	1278	2694	1987	458	338	233	172	118	87
Eritrea	34	11	5	2	193	60	74	23	271	84	743	231
Eswatini	14	12	271	236	1149	1001	2791	2431	2217	1931	2916	2540
Ethiopia	74	1	1098	10	7009	63	32 490	290	69 883	624	13 710	122
Gabon	76	35	2727	1255	3139	1445	2446	1126	803	370	380	175
Gambia	9	4	16	7	39	17	2622	1117	1079	460	35	15
Ghana	636	21	7245	238	18 691	614	17 197	565	8429	277	2732	90
Guinea	319	25	3452	270	2429	190	2767	217	4176	327	595	47
Guinea-Bissau	40	21	1282	667	605	315	222	116	292	152	6	3
Kenya	208	4	1754	33	8829	168	21 573	410	51 805	985	12 289	234
Lesotho			2	1	254	120	793	373	1088	512	440	207
Liberia	57	12	239	48	760	154	234	47	347	70	163	33
Madagascar	108	4	687	25	4548	169	8984	333	3014	112	373	14
Malawi	16	1	268	14	2213	119	2917	157	614	33	555	30
Mali	123	6	1140	58	1045	53	397	20	2057	105	2328	118
Mauritania	7	2	523	116	4988	1102	1387	306	2454	542	4283	946
Mauritius	324	256	11	9	8	6	3	2	159	126	22	17
Mozambique	21	1	233	8	1014	33	2127	70	12 375	408	2872	95
Namibia	16	6	8	3	936	375	5070	2032	8447	3386	10 068	4036
Niger	570	25	388	17	141	6	73	3	414	18	1622	71
Nigeria	343	2	9819	49	23 454	117	18 611	93	15 611	78	19 672	98
Rwanda	127	10	243	19	1046	83	1673	132	2859	226	2435	193
Sao Tome and Principe	4	19	479	2227	253	1176	156	725	104	484	18	84
Senegal	301	18	3344	205	4598	282	4706	289	3158	194	3033	186
Seychelles					95	973	29	297	37	379	95	973
Sierra Leone	10	1	851	109	790	101	341	44	421	54	147	19
South Africa	2272	39	30 411	519	265 609	4536	311 481	5319	182 526	3117	265 262	4530
South Sudan	4	0	1143	103	1006	91	354	32	611	55	440	40
Togo	77	10	365	45	289	36	546	68	1720	213	636	79
Uganda	54	1	399	9	590	13	1319	30	19 047	430	14 102	319
Zambia	45	3	1034	58	1284	72	8719	488	6583	369	3060	171
Zimbabwe	18	1	159	11	887	61	4866	332	4199	287	3738	255
African region	10 397	10	92 541	90	405 823	393	495 214	479	498 040	482	404 711	392

Nb, number of; CAR, Central African Republic; DRC, Democratic Republic of Congo.

a Data in this table may be slightly different to those publicly available on the World Health Organization website due to reporting delays.

**Fig. 3.**
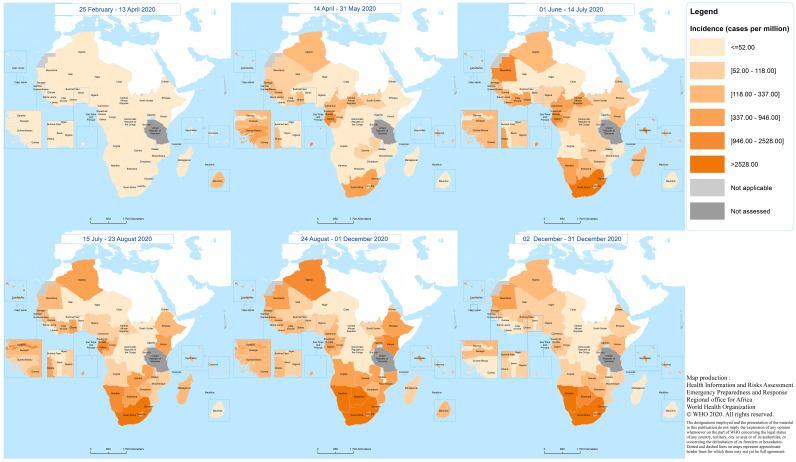
Geographical distribution of the COVID-19 incidence by period (from 25 February to 31 December 2020) in the WHO African region.

Figure 3 shows the geographical distribution of incidence rate by period from 25 February to 31 December 2020.

### Risk factors associated with high COVID-19 cumulative incidence as of 31 December 2020

Table 2 shows the association between the cumulative COVID-19 incidence and potential risk factors using the non-adjusted and adjusted negative binomial regression analyses.

**Table 2.** Multivariable analysis of factors associated with the high cumulative COVID-19 incidence in the WHO African region as of 31 December 2020

Explanatory variables	Mean value ± s.d.	Crude *β* coefficient (95% CI)	Adjusted *β* coefficient (final model) (95% CI)
% Population aged 15–64 years	56.6 ± 4.8	0.212 (0.154 to 0.275)***	0.174 (0.111 to 0.239)***
Percentage of urban population	56.6 ± 17.4	−0.014 (−0.032 to 0.004)	−0.011 (−0.002 to −0.0008)*
Population density	111.8 ± 139.5	−0.002 (−0.005 to −0.0005)*	−0.001 (−0.0003 to 0.0002)
Universal health service coverage index	56.6 ± 4.8	0.081 (0.061 to 0.102)***	
Health expenditure (per cent of growth domestic product)	5.7 ± 2.2	0.097 (−0.089 to 0.288)	−0.070 (−0.157 to 0.021)
Number of SARS-CoV2 tests performed per 10 000 population	406.9 ± 626.5	0.016 (0.001 to 0.002)***	0.0006 (0.00009 to 0.0012)**
Prevalence of diabetes (%)	5.6 ± 2.2	0.416 (0.224 to 0.621)***	
Prevalence of obesity (%)	9.3 ± 6.0	0.171 (0.017 to 0.229)***	
Crude death rate (per 10 000 population)	56.6 ± 4.8	−0.148 (−0.324 to 0.268)	
HIV infections incidence (per 1 000 population)	1.7 ± 2.2	0.207 (0.059 to 0.383)**	0.089 (−1.124 to 0.197)
Tuberculosis incidence (per 1 00 000 population)	218.4 ± 160.2	0.002 (−0.0007 to −0.005)**	
Mortality rate attributed to exposure to unsafe WASH services (per 100 000 population)	38.9 ± 23.0	−0.042 (−0.051 to −0.032)**	
Density of nursing and midwifery personnel (per 10 000 population)	25 (54.3)	0.074 (0.041 to 0.109)***	

*P < 0.05; **P < 0.01; ***P < 0.001; s.d., standard deviation; CI, confidence interval. Likelihood ratio test: *χ*^2^ = 21.1, *P* = 0.58.

In the final model, an increasing percentage of urban population (*β* = −0.011, *P* = 0.04) was associated with low cumulative incidence while an increasing number of cumulative SARS-CoV-2 tests performed per 10 000 population (*β* = 0.0006, *P* = 0.006) and the proportion of the population aged 15–64 years (adjusted *β* = 0.174, *P* < 0.0001) were associated with high COVID-19 cumulative incidence.

## Discussion

In the WHO African region, as of 31 December 2020, South Africa, Ethiopia, Algeria, Kenya and Nigeria were the countries that reported the highest cumulative number of COVID-19 confirmed cases while only South Africa was among the five countries with the highest cumulative incidence (Cape Verde, South Africa, Namibia, Eswatini and Botswana). The disease incidence (cumulative or for a specific period) is one of the metrics used for assessing infectious disease risk at national or subnational levels [13, 24, 25]. The difference in incidence between countries in the WHO African region reflects not only the heterogenicity and diversity of countries in the region, but may also reflect the varying response interventions implemented by these countries in accordance with the guidance provided by WHO at regional and global levels [26]. The COVID-19 pandemic has been assigned the highest level of operational response required by the WHO as per its emergency response framework [12]. In such a situation, the WHO support to Member States is led by regional offices with assistance from the WHO headquarters [12]. Since all 47 countries of the WHO African region are affected by the COVID-19 pandemic, monitoring the cumulative incidence which takes into account the size of the population can serve as the basis for defining priority countries for the technical and financial support from the WHO AFRO.

The COVID-19 incidence in countries can be influenced by a number of risk factors. In this study, countries with a high proportion of the population aged 15–65 years were associated with high COVID-19 cumulative incidence. Although the COVID-19 incidence is known to be higher in non-elderly populations especially in developing countries, the younger population is considered by several authors as one of the reasons for low infection rates in Africa [27, 28]. Indeed, Sub-Saharan African countries have a younger demographic structure, with only 3% over the age of 65 years, compared with 23% in Italy [29]. Our results suggest that despite the low number of COVID-19 cases reported in the African region compared to other regions in the world [4], within countries, those with a higher proportion of young population recorded higher COVID-19 incidence. According to Diop *et al*. [27], the large youth population in Sub-Saharan Africa may lead to more infections but most of these infections will be asymptomatic or mild and a proportion of them will remain undetected. Asymptomatic persons seem to account for approximately 40–45% of SARS-CoV-2 infections and can transmit the virus to others for an extended period, perhaps longer than 14 days [30, 31].

A modelling study performed by Diop *et al*. [27] showed that the presence of rural population may be a factor that limits the spread and severity of COVID-19 in Africa. Similarly, an analysis of the time-space geographies of COVID-19 infection focusing on Nigeria suggests that high population densities enhance the spread of COVID-19 [32]. In this study, no association between COVID-19 incidence and population density was found, and the proportion of urban population was negatively associated with the reported COVID-19 cumulative incidence. Our results suggest that countries whose populations are not concentrated in urban areas are more susceptible to the spread of the virus. In most countries in the African region, urban areas are the epicentres of the epidemic, accounting for the vast majority of the confirmed COVID-19 cases [33]. According to Ogunkola *et al*. [34], most attention and implementation of responses to COVID-19 have taken place in urban areas in Africa. Major challenges related to the COVID-19 response in rural areas in Africa include limited access to these areas due to poor road networks, which may hamper the possibility of providing resources and manpower, shortage of healthcare workforce, poor health facilities and limited access to COVID-19 diagnostic services [34]. A concentrated outbreak, especially in major cities, makes containment and response measures easier to implement, especially in a context of limited capacity and scarce resources. Unlike our study, Gupta *et al*. [35] found that the number of cases per million was correlated with the percentage of urban population in 17 countries in Europe and America. Gupta *et al*. [36] found a positive association between urbanisation and COVID-19 incidence in India.

In this study, the cumulative incidence of COVID-19 was positively associated with the cumulative number of SARS-CoV-2 tests performed per 10 000 population. In the context of community transmission, it is known that the more SARS-CoV-2 tests are performed the more cases are detected. Testing capacity is a major limiting factor in assessing the true extent of SARS-COV-2 transmission, as countries tend to restrict testing to individuals that meet specifically narrowed criteria [37, 38]. While countries in the WHO African region appear to be experiencing much lower rates of COVID-19 compared to countries in other WHO regions, their significantly lower testing capacity may grossly underestimate incidence rates [37]. Such correlations underscore not only the importance and centrality of testing in detection, monitoring and control of the pandemic, but also the need for Member States in the WHO African region to widely scale up testing capacity.

Our study did not find an association between the cumulative COVID-19 incidence and the prevalence or incidence of comorbidities such as diabetes, obesity, HIV infections and tuberculosis. Comorbidities are known to be risk factors for severe illness and mortality from COVID-19 [39, 40].

No association was found between high COVID-19 cumulative incidence and health and economic indicators such as health expenditure and universal health service coverage index. However, such lack of association may be related to the nature of the study, which considers only national reported figures, since subnational data are generally not available. The effect of health systems and the level of income on COVID-19 incidence may have been masked by the simultaneous nature of the COVID-19 outbreak across the region, which has universally suffered from overwhelmed health systems and response capacities.

## Limitations

In this study, we estimated the reported cumulative COVID-19 incidence assuming that repeated positive laboratory confirmations from the same individual are not included in the case count. The incidence for each country was calculated based on reported cases, which included only confirmed cases as Member States are not reporting probable cases. Given the low testing capacity in most countries of the WHO region, there is likely underdetection of cases leading to underestimation of the reported incidence. Further investigations are needed to better estimate attack rates as seroprevalence data become available for most Member States.

Regarding the selected risk factors, we used the most recent available data, most of which were not from the same year as the data used to calculate the cumulative COVID-19 incidence. Some data used were sourced from publicly available repositories and therefore subject to the limitations of their sampling, study design and data collection processes. Further, some of the risk factor metrics used, such as HIV incidence, TB incidence, mortality rate attributed to exposure to unsafe WASH services, may have been influenced by PHSM implemented by each Member State. Only national data were used for risk factors as subnational data are not available in most countries. This may have masked the association between the cumulative incidence and some metrics used as risk factors in this study. The interpretation of the results presented here should take these limitations into account.

## Conclusion

The African region continues to be affected by the COVID-19 pandemic with disparities in disease incidence. Our study showed the relationship between the cumulative COVID-19 incidence and the number of tests performed per 10 000 population, the proportion of population aged 15–64 years and the proportion of urban population. In a context of limited testing capacities and overwhelmed health systems, the findings of this study highlight the need for countries to not only increase and decentralise their testing capacities, but also to adjust testing strategies to target those most at risk in the population. The rapid control of the pandemic, even with the potential for vaccine distribution, will rely on country capacity to detect and rapidly isolate all cases. Countries need to continuously monitor the COVID-19 incidence at sub-national level to adjust public health and social measures in areas with increased incidence and ongoing widespread community transmission. More generally, our findings highlight the need for developing a culture of data management and use for strategic decision-making as part of preparedness and response to public health emergencies.

## Data availability statement

The data that support the findings of this study are available on request from the corresponding author.
